# Lymphœdème compliqué de papillomatose verruqueuse

**DOI:** 10.11604/pamj.2018.31.251.16166

**Published:** 2018-12-28

**Authors:** Fatima-zahra Agharbi

**Affiliations:** 1 Centre Hospitalier Régional Tétouan, Tétouan, Maroc

**Keywords:** Lymphœdème, papillomatose, complication, Lymphedema, papillomatosis, complication

## Abstract

Limb lymphedemas are due to a malfunction of the lymphatic system responsible for lymph stasis in the interstitial tissue and secondarily to an increase in the volume of the affected limb. They are divided into primary lymphedema (PL) and secondary lymphedema (SL). SLs develop most frequently in lower limbs after breast cancer treatment while lymphedemas are either secondary (iatrogenic or infectious) or primitive, most often sporadic, sometimes familial or they can be part of syndromes resulting in malformations and/or more complex genetics in upper limbs. The diagnosis of lymphedema is essentially based on clinical examination. Erysipelas are the main complication of lymphedema. Other abnormalities are often visible: yellowish skin and nails, lymphangiectasias, keratotic papules with papillomatosis, lichenified plaques. The main differential diagnosis in patients with limb lymphedema is lipoedema, defined as body fat distribution from the hips up to the ankles and affecting almost exclusively obese women. We report the case of a 30 year old man with left lower limb lymphedema occurred at puberty complicated by verrucous papillomatosis. It is a late-revelation congenital lymphedema.

## Image en médecine

Les lymphœdèmes des membres (LOM) sont dus à un dysfonctionnement du système lymphatique responsable d'une stase de la lymphe dans les tissus interstitiels et secondairement d'une augmentation de volume du membre atteint. On les classe en lymphœdèmes primaires (LOP) et lymphœdèmes secondaires (LOS). Au niveau du membre supérieur, les LOS après traitement d'un cancer du sein sont les plus fréquents; au niveau du membre inférieur, les LO sont soit secondaires (iatrogéniques ou infectieux), soit primitifs le plus souvent sporadiques, parfois familiaux ou peuvent faire partie des syndromes malformatifs et/ou génétiques plus complexes. Le diagnostic de LO est essentiellement clinique. L'érysipèle reste la principale complication des LO, d'autres anomalies sont fréquemment visibles: aspect jaunâtre de la peau et des ongles, lymphangiectasies, papules kératosiques avec papillomatose, plaques lichénifiées. Le principal diagnostic différentiel des LOM est le lipœdème, défini par une répartition anormale des graisses allant des hanches jusqu'aux chevilles et atteignant presque exclusivement les femmes obèses. Nous rapportons l'observation d'un homme de 30 ans qui présente depuis la puberté un lymphœdème du membre inferieur gauche compliqué par une papillomatose verruqueuse. Il s'agit d'un lymphœdème congénital à révélation tardive.

**Figure 1:**
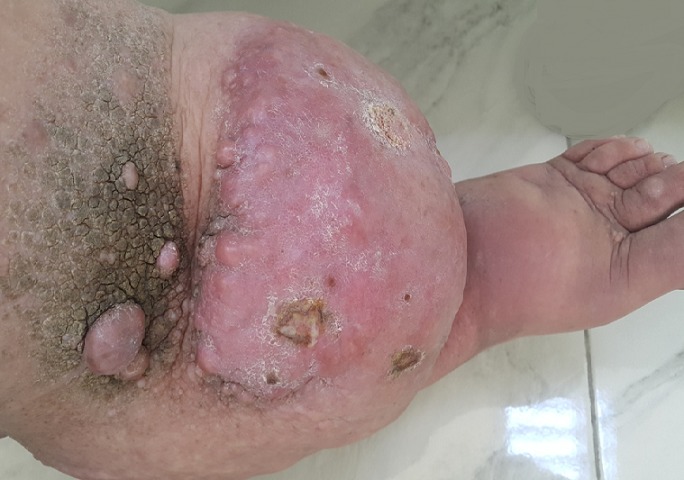
lymphœdème du membre inferieur gauche compliqué par une papillomatose verruqueuse

